# Surgery

**Published:** 1906-03

**Authors:** J. Lacy Firth


### SURGERY.

**The Indications and Results of Nephrectomy, especially for Tubercular Disease of the Kidney.**—This subject was discussed in a thorough manner at the last Congress of the German Surgical Society in April, 1905. The discussion was opened by Th. Rovsing, of Copenhagen, and it is an abstract of the remarks of that surgeon which we propose to place before our readers.

Rovsing[4] points out that as the first condition which renders the removal of a kidney justifiable is the previous demonstration of the existence of a second kidney capable of performing its functions, so in recent discussions the subject chiefly considered

1 *München. Med. Wchnschr.*, May 16th, 1905; *Med. Electrol. and Radiol.*, July, 1905.

2 *Berl. Klin. Wchnschr.*, May 15th, 1905. 3 *Archi. Röntg. Ray*, March, 1905.

4 *Zentralb. f. Chir.*, Nr. xxx., Brilage, 1905.

has been the best method of obtaining this preliminary information. But, especially in tubercular disease, other questions of importance require earnest consideration when nephrectomy is contemplated, and it was with these that the speaker first dealt. These questions are:—

First. *At what stage in tubercular disease of the kidney is nephrectomy indicated?* The views held on this question may be divided into three classes—the conservative, the moderate, and the radical. According to the conservative view, a localised tubercular deposit in the kidney may heal spontaneously, and therefore in such nephrectomy is not indicated until constitutional and internal treatment have been thoroughly tried and have proved useless and the symptoms of advancing disease are present. According to the moderate view spontaneous cure is so very rare that it should not be expected, but if on exploration the disease appears to be limited to one part of the kidney that part only should be removed, by resection. Lastly, the radical view is that the existence of tubercular disease in one kidney is of itself an absolute indication for nephrectomy.

Rovsing strongly supports the radical view, and maintains that it is unwise to leave any part of the affected kidney, because, even after splitting up the kidney as completely as possible at an exploratory operation, it is impossible to correctly define the extent of the disease.

Secondly. *How far does an extension of the disease to the bladder contra-indicate nephrectomy?*

This question is of great practical importance, because a large proportion of patients first seek medical advice when the symptoms of bladder implication are manifest. It is well established that limited tubercular deposits in the bladder about the opening of the ureter on the affected side do not contra-indicate nephrectomy, and that such deposits heal up after the operation. Until recently the case has been quite different when there has been widespread disease of the bladder, and nephrectomy has then often failed to relieve the symptoms or to prolong life more than a few months.

Rovsing states that formerly in such cases he obtained bad results after nephrectomy, in spite of the subsequent use of sublimate and pyrogallic acid injections, curetting, cauterisation, and even treatment with Finsen or Röntgen rays applied through specially constructed tubes. Latterly, however, he has obtained much better results, which he attributes to treatment by irrigation of the bladder with 5 per cent. carbolic acid solution. In seven out of eighteen cases of extensive bladder disease treated by the methods first mentioned no relief was obtained, but in ten of the remaining cases in which the carbolic method was used a cure followed. He therefore no longer regards extensive bladder disease as a contra-indication to nephrectomy.

Thirdly. *To what extent does tuberculosis in the genital organs*

*contra-indicate nephrectomy when associated with one-sided kidney disease?* The view has been frequently maintained that nephrectomy is contra-indicated when there is coincident tuberculosis of the genital organs. The author most strongly supports the opposite view. In cases of genital tuberculosis he carefully examines the patient for evidence of renal tuberculosis, and removes the kidney if one kidney only is implicated. Among twenty-four men upon whom the author performed nephrectomy there were ten—*i.e.* 41 per cent.—with genital tuberculosis, four of the patients had this on both sides, six on one side. Orchidectomy or extirpation of the epididymis was usually performed at the same sitting as the nephrectomy. All the patients excepting one were cured, and that one was operated upon under a misconception as to the results of ureteral catheterisation, and had bi-lateral kidney disease.

Fourthly. *Is it possible, and, if so, how is it possible, to determine that a second kidney is present and capable of properly performing its functions?* Even after the introduction of catheterising cystoscopes rendered the collection of the urine from each kidney feasible, other difficulties opposed the solution of the above problem. Cases occur in which a much diseased kidney secretes clear urine free from albumin, for example in cirrhosis of the kidney, cystic kidney, hydronephrosis, non-malignant tumours of the kidney. In other cases the urine from the kidney may contain albumin and pus and yet be able to take up the functions of both organs if the other one be removed. How can one in such cases avoid performing nephrectomy in unsuitable cases, or, what is worse, neglect to perform the operation where it is necessary it should be performed for the salvation of the patient?

Opinions still widely differ as to the value of the various methods of determining the functional capacity of the kidneys which have been brought forward. The principle common to all the methods so far introduced has been the estimation of the functional capacity of a kidney by measuring the work it performs, especially the excretion of certain substances. The following are some of the methods of this kind which have been tried:—

(1) Estimation of the quantity of urea excreted. (2) Estimation of the rapidity and intensity with which methyl-blue is excreted after being injected into a muscle (Achard). (3) Cryoscopy, the determination of the freezing-point of the blood and urine, from which, according to Koranyi, the excretory powers of the kidneys may be inferred. (4) The phloridzin test, which depends upon the rapidity and intensity with which the kidney excretes sugar after the injection of phloridzin subcutaneously.

The methylene-blue test has not proved reliable, since even highly degenerated kidneys may excrete the colouring matter easily. Volcker and Joseph's indigo-carmine test is better, as it colours the urine much more intensely, but that substance also gives unreliable results.

The estimation of urea, cryoscopy and the phloridzin tests have been widely accepted as giving reliable information of the kind so much desired. Casper and Richter believe all these three methods are reliable, and place the phloridzin test first, cryoscopy second, and the estimation of urea third in degree of reliability. Kümmell considers that a blood freezing-point of $-0.6°$ C. shows that both kidneys are diseased and absolutely contra-indicates nephrectomy. Casper admits that the phloridzin test sometimes gives irrelevant results, but Kümmell declares that cryoscopy has never led him into error.

Rovsing has tried all three methods, but has come to quite other conclusions as to their reliability than the writers just mentioned. With regard to the estimation of urea, he has found that if the quantity of urea excreted by one kidney is normal, then that organ has always good excretory power; but the reverse does not hold true, for a diminished excretion of urea does not show that the organ in question is so incapable of performing its functions that the other kidney cannot be safely extirpated. In thirty-one of Rovsing's 112 nephrectomies the urine contained very little urea, yet the results of nephrectomy were good. Similarly with the phloridzin test, the author never found that a kidney which excreted the sugar well was insufficient, but in nine cases nephrectomy was performed though no sugar at all was excreted. Eight of these cases recovered and one died. The death was not from renal insufficiency but from hemorrhage and septicity.

In fifty cases Rovsing obtained the freezing-point of the blood, and twelve times the results would have led to serious error if they had been relied upon. Errors both of a positive and of a negative kind may result from cryoscopy. In six cases nephrectomy was successfully performed, though the freezing-point of the blood was from $-0.6°$ C. to $-0.67°$ C., yet the one kidney left proved fully capable of performing the functions of the two. In six other cases the freezing-point of the blood was found normal in the presence of advanced disease of both kidneys. That Casper and Kümmell have come to different conclusions with regard to the value of cryoscopy is due, in Rovsing's opinion, to the fact that those surgeons have assumed the truth of the proposition which was to be proved—for example, the proposition that a freezing-point of the blood of $-0.6°$ C. showed that both kidneys were diseased, and that nephrectomy would therefore be fatal—and having assumed its truth, they have not tested its truth in the only reliable way, namely by performing nephrectomy when the freezing-point of the blood was low and watching the results.

Rovsing next considers the results he has obtained in his 112 nephrectomies. In fifty-two cases operated upon before

1901 the mortality was 13.2 per cent.; in sixty cases operated upon since 1901 the mortality was 3.3 per cent.

Kümmell, in discussing the value of cryoscopy of the blood, lays stress on the fact that since he has allowed himself to be guided by its results he has reduced his mortality in nephrectomy from 33 per cent. to 6.4 per cent. Rovsing's statistics show that he himself has reduced the mortality of his nephrectomies to a lower point even than Kümmell, although he has operated in spite of unfavourable findings by cryoscopy and by the phloridzin tests. Further, the two cases he has lost since 1901 were not lost through insufficiency of the remaining kidney, but from bleeding and sepsis.

Rovsing's conclusions therefore on the questions discussed above are:—

(1) That renal insufficiency as a cause of death after nephrectomy does not play the part attributed to it by Casper and Kümmell. (2) That the functional capacity of the kidneys cannot be accurately ascertained by any of the procedures which have been introduced for the purpose. All of the procedures depend upon the measurement of the work being performed by the kidneys, and it may be assumed that procedures depending upon such principles never will give satisfactory results, because a depression of renal function is by no means identical with a diminution of the capacity of the kidney to perform its functions. A normal kidney may work badly for various reasons, *e.g.* because the other kidney is diseased, and not because it is incapable of working well under more favourable conditions. (3) That recent improvements in the results of nephrectomy are not due to the introduction of the cryoscopic and phloridzin methods of estimating the renal functional capabilities. (4) Cryoscopy of the blood is of no value in considering the indications for and against nephrectomy, for it leads to errors in two directions, appearing to show bilateral disease when the disease is unilateral and vice versa. (5) The phloridzin and urea tests are reliable if positive, but not if negative, and that of the two the urea estimation is the better.

The author finally asks: "If my improved results in nephrectomy have not been obtained through the methods of determining renal functional capacity, to what methods can they be attributed? What procedures have enabled me to correctly estimate the functional capacity of the other kidney?" The answer is: "I have to thank catheterisation of the ureters and the careful chemical, microscopical and bacteriological examination of the urine collected directly from each kidney, and the importance of this is much greater than the phloridzin test and cryoscopy. If I have found the collected urine without albumin, blood and microbes, I have considered, as a rule, operative attack to be justified." If in a case of tubercular or other pyonephrosis the author finds the urine from the other kidney contains albumin, then the deciding-point for or against nephrectomy is the presence or absence of pus and microbes in that urine. If albumin only is found, then it is concluded that it is a toxic albuminuria, and determined by the disease in the pyonephrotic kidney. The latter is therefore removed as soon as possible.

Rovsing sounds a note of warning against using urine segregators instead of catheterising the ureters, for the segregator will often lead to error: for example, in the presence of bladder disease it will lead to the diagnosis of bilateral disease when the disease really is unilateral.

J. LACY FIRTH.

